# Development and validation of a primary care–accessible risk prediction model for severe pulmonary TB

**DOI:** 10.5588/ijtldopen.26.0061

**Published:** 2026-09-15

**Authors:** X. Hu, W. Shu, N. Su, C. Bei, Y. Wu, J. Lei, D. Sun, S. Gao, Y. Zhu, L. Li, J. Gao

**Affiliations:** 1 Beijing Chest Hospital, Capital Medical University/Beijing Tuberculosis and Thoracic Tumor Research Institute, GCP Administration Office, Beijing, China;; 2 Beijing Chest Hospital, Capital Medical University/Beijing Tuberculosis and Thoracic Tumor Research Institute/Clinical Center on Tuberculosis, China CDC, Beijing, China;; 3 Guangzhou Chest Hospital, Guangzhou, Guangdong, China;; 4 Changsha Central Hospital, Changsha, Hunan Province, China;; 5 Jiangxi Chest Hospital, Nanchang, China.

**Keywords:** tuberculosis, China, risk prediction model, primary care, nomogram

## Abstract

**BACKGROUND:**

China has a high TB burden and limited primary care. We therefore developed a risk prediction model using routine clinical and lab indicators to flag severe TB cases early.

**METHODS:**

In a case–control study of 2,012 TB patients (499 severe, 1,513 non-severe) from four hospitals (2012–2022), patients were split into training (n = 1,408) and validation (n = 604) sets. Univariable and multivariable logistic regression identified predictors to build a nomogram (i.e., a tool that integrates patient-specific data to predict the probability of a specific clinical event), with model performance assessed using receiver operating characteristic (ROC) curves, calibration, and decision curves.

**RESULTS:**

Multivariable analysis identified seven independent predictors: advanced age, history of TB, lymphocytopenia, monocytosis, neutrophilia, hyponatremia, and hypoalbuminemia. The area under the ROC curve (AUC) for the training set was 0.834 (95% confidence interval [CI]: 0.796–0.872), with a sensitivity of 89.0% and specificity of 63.4%; the validation set AUC was 0.807 (95% CI: 0.746–0.867), with sensitivity 82.0% and specificity 71.7%. The calibration curve showed high consistency between predicted probabilities and actual observations. Decision curve analysis demonstrated clinical net benefit of the model within the threshold probability range of 5%–95%.

**CONCLUSION:**

This model, based on routine indicators, effectively identifies high-risk severe TB cases, offering a practical and cost-efficient tool for early patient stratification in primary care.

TB is caused by the bacillus *Mycobacterium tuberculosis*, which is transmitted when individuals with active TB expel bacteria into the air. In 2024, an estimated 10.7 million people worldwide developed TB, and the disease caused approximately 1.23 million deaths, making it the leading cause of death from a single infectious agent. As one of the 30 high-burden countries, in 2024, China reported around 696,000 newly diagnosed TB cases with an estimated 25,000 deaths among HIV-negative TB patients.^1^ A subset of patients progress to critical illness, complicated by multi-organ failure, respiratory failure, septic shock, or acute kidney injury, and require intensive care; in this group mortality is higher than in ordinary TB. Even in severe TB patients who receive treatment and care in the TB Intensive Care Unit (TB-ICU), case fatality remains as high as 60%–80%.^2–5^

China has a large patient population but limited primary health care resources. Therefore, a risk prediction model for progression to severe disease that can be widely implemented at the primary-care level is urgently needed to enable patients hierarchical management and timely referral of high-risk cases to higher-level hospitals. Research has demonstrated that a prediction model incorporating diabetes, the systemic inflammation response index (SIRI), vascular endothelial growth factor (VEGF), and interleukin-6 (IL-6) can reliably provide an early estimate of the risk of developing severe pulmonary TB (PTB).^6^ However, SIRI, VEGF, and IL-6 are not routinely measured in primary institutions, preventing the model’s real-world application. At present, an effective screening tool suitable for primary care settings is lacking; consequently, a substantial proportion of high-risk patients miss the best time for referral, and life-saving treatment is delayed. Therefore, developing a primary-level risk prediction model for PTB progression has become essential.

This study aims to construct a practical and reliable early risk-assessment tool (a nomogram) based on investigations that can be readily obtained at the primary-care level, thereby assisting primary-level hospitals in promptly identifying severe PTB cases and initiating referral, ultimately reducing TB-related mortality.

## METHODS

This multicentre case–control study enrolled TB patients treated at four hospitals between 2012 and 2022, including Beijing Chest Hospital, Guangzhou Chest Hospital, Jiangxi Chest Hospital, and Changsha Central Hospital. These centres receive referrals from lower-level facilities. Patients were classified according to the presence or absence of severe disease. Severe PTB was defined by the fulfilment of at least one of the following criteria: 1). Peripheral oxygen saturation ≤ 93% while breathing room air at rest; 2) Arterial oxygen tension/inspiratory oxygen fraction ratio (PaO_2_/FiO_2_) ≤ 300 mm Hg, or arterial oxygen tension (PaO_2_) ≤ 60 mm Hg while breathing room air at rest; 3) Chest imaging demonstrating bilateral pulmonary lesions involving > 50% of the total lung parenchyma; 4) Documented miliary TB or TB meningitis. Patients who did not meet any of the above criteria were allocated to the control group.

### Data collection

Upon admission, baseline demographic characteristics (including age, gender, body mass index [BMI], smoking and alcohol use history, and comorbidities such as fever, diabetes and hypertension) were systematically recorded. Laboratory data comprised: 1) haematologic indices: haemoglobin, white blood cell, platelet, lymphocyte, monocyte, and neutrophil counts; 2) biochemical parameters: alanine aminotransferase, aspartate aminotransferase (AST), alkaline phosphatase (ALP), total and direct bilirubin (TBil/DBil), creatinine, sodium (Na), potassium (K), albumin (ALB), glucose, and C-reactive protein (CRP). Additionally, the presence of invasive bacterial or fungal infections was documented.

### Statistical analysis

The data cleaning procedures were performed in accordance with the categories and inherent characteristics of the collected data. During the analysis, missing data that could not be supplemented were directly excluded from subsequent analyses. All statistical analyses were performed using SPSS version 26.0 and R version 4.3.1. Continuous variables were tested for normality. Normally distributed variables are presented as mean ± standard deviation and compared using the independent-samples *t* test, whereas non-normally distributed variables are presented as median (interquartile range) and compared with the Mann–Whitney *U* test. Categorical variables are expressed as counts (percentages) and compared using the *χ*^2^ test or Fisher’s exact test, as appropriate. Variables in univariable analyses (*P* < 0.05) were entered into a multivariable logistic regression model. A nomogram based on logistic regression was constructed, and the diagnostic performance of the model was evaluated using the receiver operating characteristic (ROC) curve. Two-sided *P* values < 0.05 were considered statistically significant.

### Ethical statement

This clinical study was conducted in accordance with the principles of the Declaration of Helsinki. This retrospective study was approved by the Ethics Committee of Beijing Chest Hospital, Capital Medical University. Given the retrospective design and use of anonymised data extracted from electronic medical records, the requirement for individual informed consent was waived by the ethics committee.

## RESULTS

This study enrolled a total of 2,012 patients with TB, including 1,513 non-severe cases and 499 severe cases. The number of patients enrolled at each centre is shown in Supplementary Data Table S1. Patients were randomly assigned in a 7:3 ratio to the training set (n = 1,408) and validation set (n = 604) using a random seed of 1,234. Patients were stratified into severe and non-severe groups based on the presence or absence of severe TB criteria at hospital admission (Supplementary Data Table S2).

The training set comprised 1,408 patients, including 356 in the severe group and 1,052 in the non-severe group. Comparison of baseline characteristics between the two groups revealed statistically significant differences in gender, age, BMI, hypertension, diabetes mellitus, invasive fungal infection, alcohol use, smoking, history of TB, and multiple laboratory parameters (white blood cell, haemoglobin, platelet, lymphocyte, monocyte, and neutrophil counts; AST, ALP, DBil, Na, K, Glucose, ALB levels, and CRP) (*P* < 0.05). Details are presented in Supplementary Data Table S3. Multivariable logistic regression analysis identified seven independent predictors, including advanced age, history of TB, lymphocytopenia, monocytosis, neutrophilia, hyponatremia, and hypoalbuminemia.

**Figure 1.**
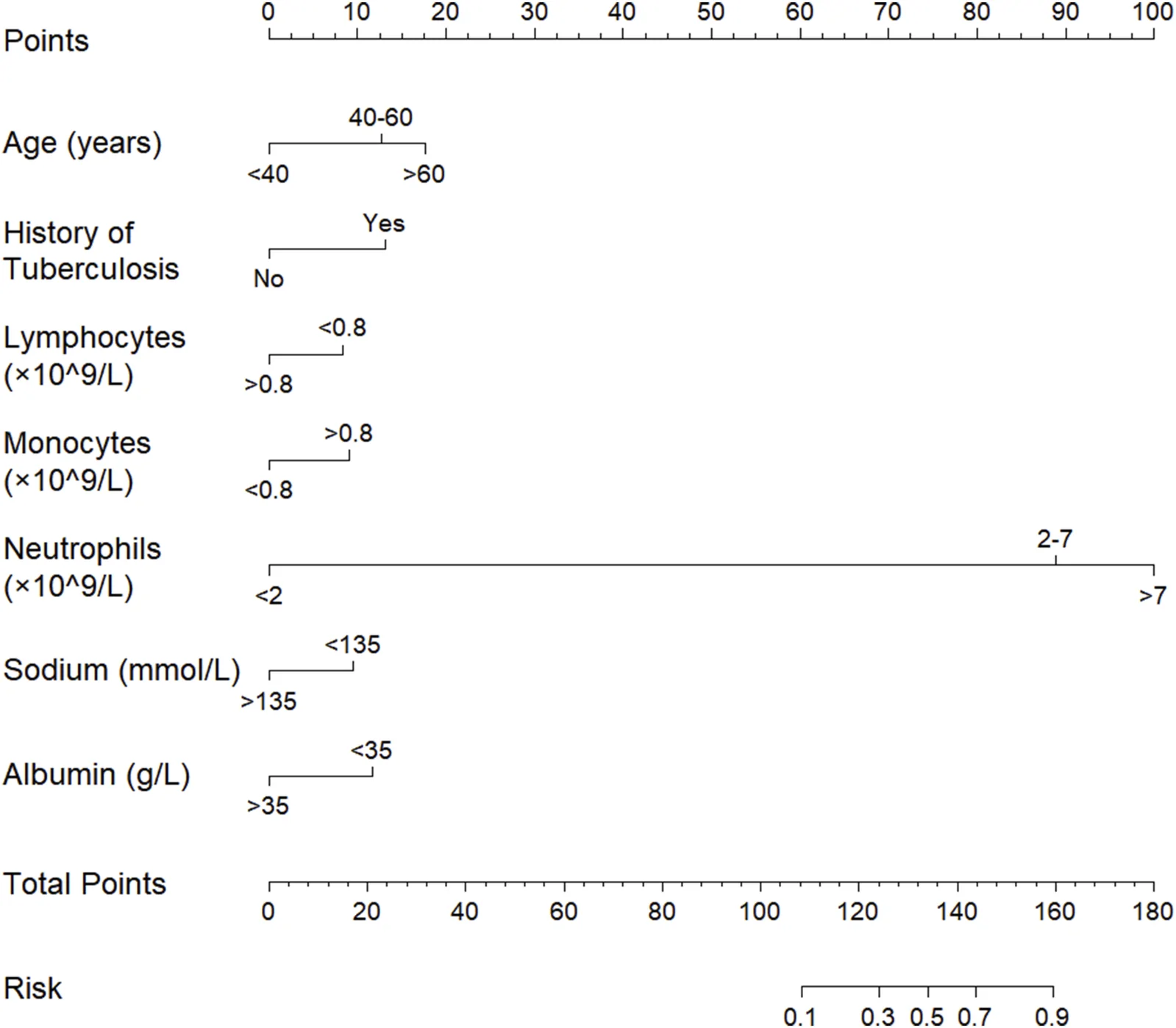
Nomogram to predict the outcomes of patients with severe pulmonary TB. To use the nomogram, locate the patient’s value on each variable axis and draw a vertical line upward to the ‘Points’ axis to determine the score for each variable. Sum the points for all seven variables to obtain the ‘Total Points’. Then, draw a vertical line downward from the ‘Total Points’ axis to the ‘Risk’ axis to estimate the probability.

**Figure 2.**
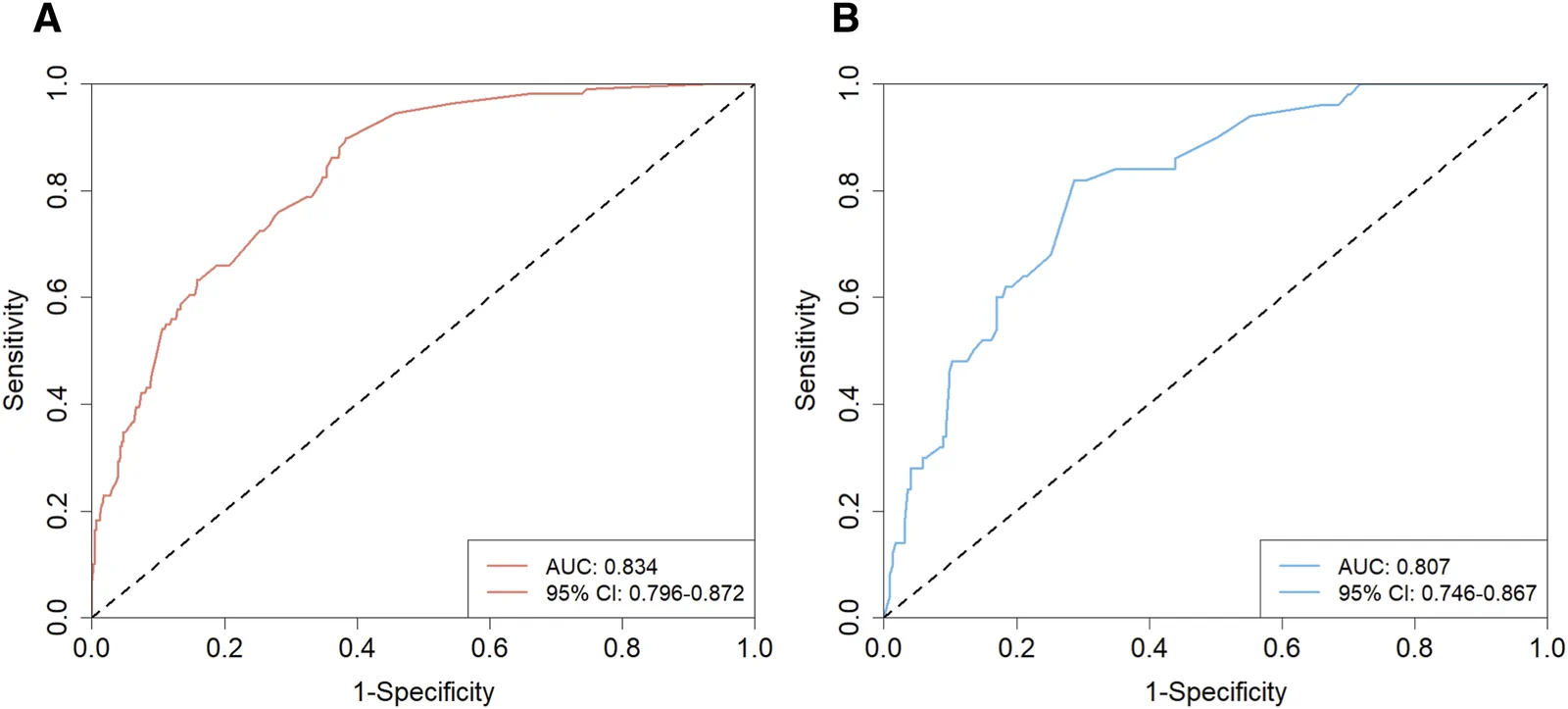
Receiver operating characteristic(ROC) curves of the nomogram for predicting severe pulmonary TB in (**A**) the training set and (**B**) the validation set. AUC = area under the ROC curve; CI = confidence interval.

A nomogram prediction model was constructed using R software, incorporating seven variables including advanced age, history of TB, lymphocytopenia, monocytosis, neutrophilia, hyponatremia, and hypoalbuminemia., as shown in Figure 1. ROC curve analysis demonstrated that the model achieved an area under the ROC curve (AUC) of 0.834 (95% confidence interval [CI]: 0.796–0.872) in the training cohort, with sensitivity and specificity of 89.0% (95% CI: 64.2%–96.3%) and 63.4% (95% CI: 53.8%–86.9%), respectively. In the internal validation cohort, the AUC was 0.807 (95% CI: 0.746–0.867), with sensitivity decreasing to 82.0% (95% CI: 66.0%–92.0%) and specificity increasing to 71.7% (95% CI: 64.6%–84.8%) (see Figure 2).

**Figure 3.**
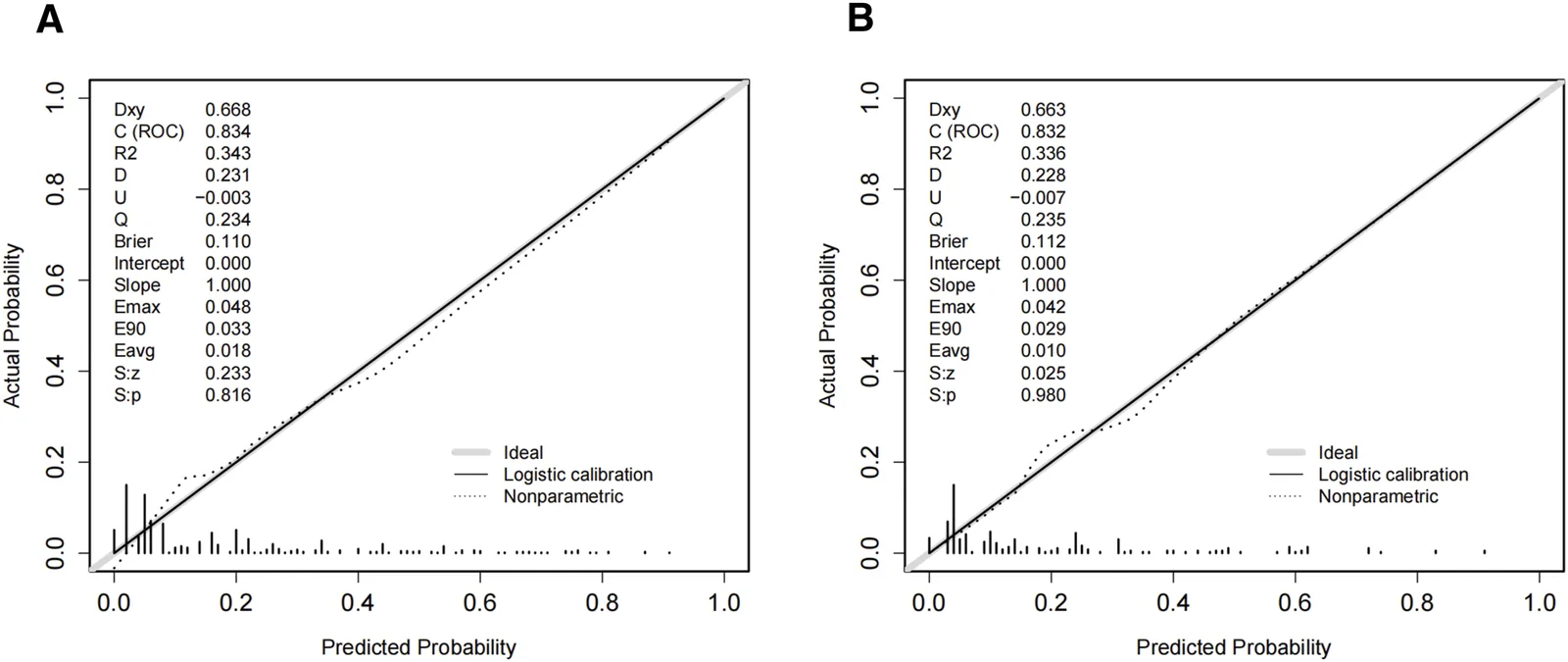
Calibration of the nomogram to predict the death in (**A**) the training set and (**B**) the validation set. ROC = receiver operating characteristic. Dxy = Somers' Dxy; R^2^ = coefficient of determination; D = discrimination index; U = unreliability index; Q = quality index; Brier = Brier score; Intercept = calibration intercept; Slope = calibration slope; Emax = maximum absolute error; E90 = 90th percentile of absolute error; Eavg = average absolute error; S:z = Spiegelhalter's Z-statistic; S:p = Spiegelhalter's *P* value.

To evaluate the predictive accuracy of the model, calibration curves for the training set (Figure 3A) and validation set (Figure 3B) were plotted using R software, with the x-axis representing predicted severe disease risk and the y-axis representing actual observed severe disease incidence. For clinical use, locate patient values on each variable axis and read upward to the Points scale to determine individual scores; sum these for Total Points (0–450); then read downward to Risk of Disease to obtain predicted probability. Clinicians can obtain an immediate quantitative estimate of severe disease risk, which may guide early intervention and resource allocation.

**Figure 4.**
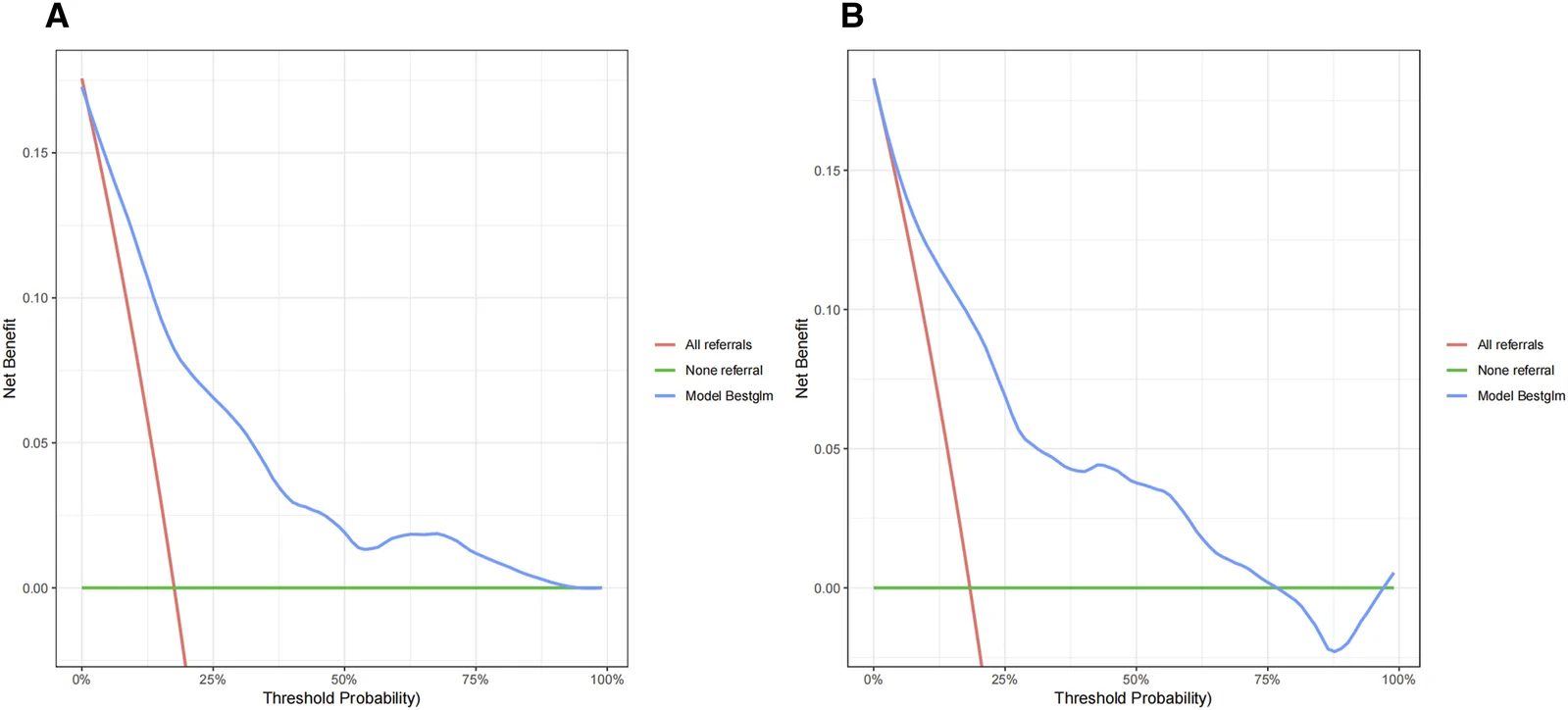
Decision curves analysis for the nomogram in (**A**) the training set and (**B**) the validation set.

The Brier score for the training set calibration curve was 0.110 (mean squared error between predicted and observed values), indicating good predictive accuracy. For the validation set, the Brier score was 0.112, demonstrating consistent calibration performance in the internal validation cohort (*P* = 0.896). The calibration curve showed good agreement with the ideal reference line (*P* = 0.816), demonstrating high consistency between predicted probabilities and actual observations. Additionally, decision curve analysis for the training set (Figure 4A) and validation set (Figure 4B) was performed using R software to evaluate the clinical benefit of the model. In the training set, the decision curve was positioned above the two extreme strategy lines of ‘treat none’ and ‘treat all’ within the threshold probability range of 5%–95%, indicating clinical benefit of the model within this interval. In the validation set, the model also demonstrated clinical utility when the threshold probability ranged from 5% to 75%.

## DISCUSSION

China remains a high-burden country for TB, where limited primary health care resources frequently delay diagnosis of severe cases, resulting in increased pre-treatment mortality.^1,7^ As patients are often transferred to tertiary hospitals only after disease progression, this study aimed to develop a prediction model applicable in primary care settings for early identification of severe TB.

While existing prognostic scores such as the Bandim TB score and Simplified TB score II focus on mortality prediction in hospitalised TB patients, this study develops a primary care–accessible tool for early identification of severe TB risk at the time of diagnosis.^8,9^ By predicting progression to severe disease – rather than mortality – our model enables preventive intervention and timely referral, potentially reducing pre-treatment mortality through early escalation of care. Using routinely available parameters – including advanced age, history of TB, lymphocytopenia, monocytosis, neutrophilia, hyponatremia, and hypoalbuminemia as independent risk factors – the model achieved an AUC of 0.834. The nomogram is designed for application at initial patient presentation to enable immediate risk stratification and guide referral decisions before disease progression. While performance was comparable to prior biomarker studies,^6,10^ this model uniquely combines the data available in primary care, offering superior generalisability and practical applicability for early risk stratification in resource-limited settings.

Clinical actions triggered by risk category: high-risk patients (>50% probability): The nomogram result triggers immediate referral to tertiary hospitals with TB-ICU capacity, hospitalisation for intensive monitoring, and supportive measures such as oxygen therapy and nutritional support. Moderate- and low-risk patients (<50% probability): Prompts referral to district-level hospitals with enhanced outpatient monitoring and patient education on warning signs requiring emergent care.

Studies have shown that advancing age is an important independent risk factor for severe TB. The incidence of active TB in individuals aged over 60 years is significantly higher than in the non-elderly population,^11^ and their risk of developing severe disease and infectiousness are also significantly increased.^12^ Reactivation of latent TB infection is most common among elderly patients, primarily attributed to the decline in immunity associated with ageing; meanwhile, age-related comorbidities often mask TB symptoms, leading to delayed diagnosis. Furthermore, the clinical manifestations of TB in the elderly are frequently atypical, further increasing the difficulty of early recognition.^13^ In China, where population ageing is accelerating, advanced age – particularly individuals over 60 years – has become a major public health challenge for the prevention and control of severe TB.

Lymphocyte-mediated cellular immunity plays a central role in anti-TB defence. TB infection can cause decreased lymphocyte counts, with the decline in CD4^+^ T cell number being inversely correlated with disease severity – lymphopenia is more pronounced in severe patients, representing an immunological hallmark of severe TB infection.^14–17^ An elevated monocyte count serves as an independent risk factor for poor prognosis in active TB and is significantly associated with impaired closure of pulmonary cavities.^18^ As prototypical innate immune cells, monocytes function as pivotal effectors and regulators of inflammatory and innate immune response.^19,20^ Furthermore, multiple transcriptomic and phenotypic studies have demonstrated that specific monocyte subsets are directly correlated with the progression and clinical activity of TB.^21–23^ Meanwhile, neutrophil response is positively correlated with TB severity; excessive neutrophil infiltration not only drives progression of pulmonary lesions but also increases the risk of secondary infection.^24^ Longitudinal studies have demonstrated that initial neutrophil levels can predict long-term lung damage, while persistent neutrophilic inflammation represents a key pathological basis for post-TB lung dysfunction.^25^ Therefore, dynamic monitoring of lymphocyte, monocyte, and neutrophil changes should be incorporated into routine clinical assessment protocols for severe TB.

We demonstrated that hyponatremia was associated with severe TB, consistent with previous findings linking hyponatremia to mortality in TB patients.^26^ TB may induce syndrome of inappropriate antidiuretic hormone secretion through invasion of the hypothalamus or via ectopic antidiuretic hormone production associated with pulmonary infection.^27,28^ Hyponatremia itself can cause a spectrum of symptoms ranging from subclinical manifestations to severe complications and has been consistently associated with adverse outcomes and increased mortality.^27,29^ Multiple studies have confirmed that hypoalbuminemia is significantly associated with an elevated risk of TB-related mortality.^30,31^ Reduced ALB levels not only indicate protein-energy malnutrition but also compromise nutrient transport and the maintenance of plasma colloid osmotic pressure.^32^ Moreover, hypoalbuminemia reflects a systemic hyperinflammatory and hypercatabolic state, resulting in diminished immune function and impaired clearance of TB.^33^ Both parameters represent crucial laboratory indicators for clinical management of severe TB.

All predictors included in this nomogram are readily obtainable through routine clinical assessments and basic laboratory tests available at primary-level health care facilities. This model requires no specialised equipment or advanced molecular diagnostics, enabling health care providers in resource-constrained settings to perform early risk stratification with minimal additional cost or training. By using routinely collected clinical data, this tool facilitates the early identification of high-risk patients, supporting timely referral decisions and potentially reducing pre-treatment mortality in severe TB cases.

This study has several limitations. First, as a retrospective analysis, it is susceptible to information bias and selection bias, and incomplete documentation of certain clinical variables may have compromised the robustness of our findings. Second, the model was developed using data from tertiary referral centres, which may limit its generalisability to primary care settings. Patients in referral centres typically represent a more severe disease spectrum; consequently, direct application of our model in primary care settings may overestimate risk, necessitating prospective validation at this level to determine the timeframe preceding severity onset during which reliable prediction can be achieved. Third, the absence of a standardised definition for severe TB may further limit model generalisability across different geographical and clinical contexts. Our criteria – respiratory failure, >50% lung involvement, and CNS disease – reflect ICU-relevant severity in the Chinese context, whereas alternative definitions emphasising sepsis, organ dysfunction, or microbiological burden are used globally. Fourth, China is a country with low HIV prevalence; therefore, this model has not been validated in high-HIV-prevalence populations, and its predictive performance may be compromised by HIV-related immunosuppression. Nevertheless, the biological plausibility of our predictors (inflammatory markers, nutritional status) suggests the model framework remains valid across settings, and future external validation studies should assess performance against locally applicable severe TB criteria and adjust prediction thresholds accordingly to ensure appropriate local calibration.

## CONCLUSION

While existing prognostic scores such as the Bandim TB score and Simplified TB score II focus on mortality prediction in hospitalised TB patients, this study addresses a distinct clinical need: early identification of severe TB risk at the primary-care level using routinely available laboratory indicators. By predicting progression to severe disease – rather than mortality – our model enables preventive intervention and timely referral, potentially reducing pre-treatment mortality in resource-limited settings.

## Supplementary Material
