# Supplementary material for: Development and validation of a primary care–accessible risk prediction model for severe pulmonary TB
Source: IJTLD Open. 2026 Sep 15;3(9):561–7. doi: 10.5588/ijtldopen.26.0061 (PMC13577203; doi:10.5588/ijtldopen.26.0061)
Supplement: Supplementary file 1 [file ijtldopen26-0061_supplementarydata1.pdf]

Table S1 Number of patients enrolled from each center.

Center	Total (n = 2012)	Non-severe (n = 1513)	Severe (n = 499)
Beijing Chest Hospital	1716 (85.29)	1315 (86.91)	401 (80.36)
Guangzhou Chest Hospital	88 (4.37)	59 (3.90)	29 (5.81)
Jiangxi Chest Hospital	142 (7.06)	93 (6.15)	49 (9.82)
Changsha Central Hospital	66 (3.28)	46 (3.04)	20 (4.01)

Table S2 Baseline characteristics of patients in the test and train sets.

Variables	Total (n = 2012)	Validation (n = 604)	Training (n = 1408)	*P*
Group, n(%)				0.444
Non-severe	1513 (75.20)	461 (76.32)	1052 (74.72)	
Severe	499 (24.80)	143 (23.68)	356 (25.28)	
Gender, n(%)				0.178
Female	676 (33.60)	216 (35.76)	460 (32.67)	
Male	1336 (66.40)	388 (64.24)	948 (67.33)	
Age, n(%)				0.749
<40	681 (34.19)	202 (33.67)	479 (34.41)	
40-59	615 (30.87)	181 (30.17)	434 (31.18)	
≥60	696 (34.94)	217 (36.17)	479 (34.41)	
BMI, n(%)				0.367
≤18.5	1190 (74.00)	361 (75.52)	829 (73.36)	
>18.5	418 (26.00)	117 (24.48)	301 (26.64)	

Fever, n(%)				0.003
No	1286 (65.08)	355 (60.17)	931 (67.17)	
Yes	690 (34.92)	235 (39.83)	455 (32.83)	
Hypertension, n(%)				0.035
No	1698 (84.39)	494 (81.79)	1204 (85.51)	
Yes	314 (15.61)	110 (18.21)	204 (14.49)	
Diabetes, n(%)				0.780
No	1504 (74.75)	449 (74.34)	1055 (74.93)	
Yes	508 (25.25)	155 (25.66)	353 (25.07)	
Invasive fungus infection, n(%)				0.754
No	1249 (83.43)	370 (83.90)	879 (83.24)	
Yes	248 (16.57)	71 (16.10)	177 (16.76)	
Invasive bacteria infection, n(%)				0.620
No	1505 (95.98)	450 (96.36)	1055 (95.82)	
Yes	63 (4.02)	17 (3.64)	46 (4.18)	
Alcohol use, n(%)				0.988
No	1435 (77.74)	430 (77.76)	1005 (77.73)	
Yes	411 (22.26)	123 (22.24)	288 (22.27)	
Smoking, n(%)				0.510
No	1219 (62.16)	372 (63.27)	847 (61.69)	
Yes	742 (37.84)	216 (36.73)	526 (38.31)	
History of tuberculosis, n(%)				0.143
No	1523 (75.85)	471 (77.98)	1052 (74.93)	
Yes	485 (24.15)	133 (22.02)	352 (25.07)	

WBC×10^9/L, n(%)				0.353
<4	1524 (76.47)	467 (78.22)	1057 (75.72)	
4-10	176 (8.83)	45 (7.54)	131 (9.38)	
>10	293 (14.70)	85 (14.24)	208 (14.90)	
HGB (g/L), n(%)				0.761
>90	1858 (93.23)	555 (92.96)	1303 (93.34)	
≤90	135 (6.77)	42 (7.04)	93 (6.66)	
PLT×10^9/L, n(%)				0.070
>100	1948 (97.74)	590 (98.66)	1358 (97.35)	
<100	45 (2.26)	8 (1.34)	37 (2.65)	
Lymphocyte×10^9/L, n(%)				0.713
⩾0.8	1661 (83.38)	495 (82.91)	1166 (83.58)	
<0.8	331 (16.62)	102 (17.09)	229 (16.42)	
Monocyte×10^9/L, n(%)				0.021
⩽0.8	1674 (84.04)	519 (86.93)	1155 (82.80)	
>0.8	318 (15.96)	78 (13.07)	240 (17.20)	
Neutrophil×10^9/L, n(%)				0.267
2-7	1526 (76.61)	470 (78.73)	1056 (75.70)	
<2	110 (5.52)	27 (4.52)	83 (5.95)	
>7	356 (17.87)	100 (16.75)	256 (18.35)	
ALT(U/L), n(%)				0.414
≤40	1772 (89.72)	527 (88.87)	1245 (90.09)	
>40	203 (10.28)	66 (11.13)	137 (9.91)	
AST(U/L), n(%)				0.959

≤40	1729 (87.41)	518 (87.35)	1211 (87.44)	
>40	249 (12.59)	75 (12.65)	174 (12.56)	
ALP(U/L), n(%)				0.298
≤150	1728 (93.76)	507 (92.86)	1221 (94.14)	
>150	115 (6.24)	39 (7.14)	76 (5.86)	
TBil(μmol/L), n(%)				0.730
≤20.5	1820 (92.11)	549 (92.42)	1271 (91.97)	
>20.5	156 (7.89)	45 (7.58)	111 (8.03)	
DBil(μmol/L), n(%)				0.347
≤7	1838 (93.06)	547 (92.24)	1291 (93.42)	
>7	137 (6.94)	46 (7.76)	91 (6.58)	
Cre(μmol/L), n(%)				0.269
≤104	1899 (96.35)	570 (95.64)	1329 (96.65)	
>104	72 (3.65)	26 (4.36)	46 (3.35)	
Na(mmol/L), n(%)				0.380
⩾135	1623 (83.02)	494 (84.16)	1129 (82.53)	
<135	332 (16.98)	93 (15.84)	239 (17.47)	
K(mmol/L), n(%)				0.544
⩾3.5	1757 (89.83)	531 (90.46)	1226 (89.55)	
<3.5	199 (10.17)	56 (9.54)	143 (10.45)	
Glucose(mmol/L), n(%)				0.921
⩽6.1	1321 (69.16)	394 (69.00)	927 (69.23)	
>6.1	589 (30.84)	177 (31.00)	412 (30.77)	
ALB(g/L), n(%)				0.295

≥35	1197 (60.58)	350 (58.82)	847 (61.33)	
<35	779 (39.42)	245 (41.18)	534 (38.67)	
CRP(mg/L), n(%)				0.651
≤5	449 (26.73)	132 (25.98)	317 (27.05)	
>5	1231 (73.27)	376 (74.02)	855 (72.95)	

BMI:Body Mass Index; WBC:white blood cell; HGB:hemoglobin; PLT:platelet; ALT:alanine aminotransferase; AST:Aspartate aminotransferase; ALP:Alkaline phosphatase;TBil:Total Bilirubin;DBil:Direct bilirubin; Cre:Creatinine;Na:sodium;K:potassium; ALB:albumin;CRP:C-reactive protein

Table S3 Univariable and Multivariable logistic regression analysis of factors in severe and non-severe tuberculosis patients

Variables	Total (n = 1408)	Non-severe (n = 1052)	Severe (n = 356)	Univariable analysis		Multivariable analysis	
				P	OR (95%CI)	*P*	OR (95%CI)
Gender, n(%)							
Female	460 (32.67)	366 (34.79)	94 (26.40)		1.00 (Reference)		1.00 (Reference)
Male	948 (67.33)	686 (65.21)	262 (73.60)	0.004	1.49 (1.14 ~ 1.94)	0.248	0.68 (0.36 ~ 1.30)
Age, n(%)							
<40	479 (34.41)	420 (40.35)	59 (16.81)		1.00 (Reference)		1.00 (Reference)
40-59	434 (31.18)	327 (31.41)	107 (30.48)	<.001	2.33 (1.64 ~ 3.30)	<.001	3.10 (1.59 ~ 6.05)
≥60	479 (34.41)	294 (28.24)	185 (52.71)	<.001	4.48 (3.22 ~ 6.22)	<.001	4.31 (2.14 ~ 8.68)
BMI, n(%)							
≤18.5	829 (73.36)	696 (75.41)	133 (64.25)		1.00 (Reference)		1.00 (Reference)
>18.5	301 (26.64)	227 (24.59)	74 (35.75)	0.001	1.71 (1.24 ~ 2.35)	0.597	1.16 (0.66 ~ 2.04)
Fever, n(%)							
No	931 (67.17)	711 (68.37)	220 (63.58)		1.00 (Reference)		
Yes	455 (32.83)	329 (31.63)	126 (36.42)	0.101	1.24 (0.96 ~ 1.60)		
Hypertension, n(%)							
No	1204 (85.51)	912 (86.69)	292 (82.02)		1.00 (Reference)		1.00 (Reference)
Yes	204 (14.49)	140 (13.31)	64 (17.98)	0.031	1.43 (1.03 ~ 1.97)	0.219	0.60 (0.27 ~ 1.35)
Diabetes, n(%)							
No	1055 (74.93)	804 (76.43)	251 (70.51)		1.00 (Reference)		1.00 (Reference)
Yes	353 (25.07)	248 (23.57)	105 (29.49)	0.026	1.36 (1.04 ~ 1.77)	0.465	1.31 (0.63 ~ 2.73)
Invasive fungus infection,							
No	879 (83.24)	688 (86.32)	191 (73.75)		1.00 (Reference)		1.00 (Reference)

Yes	177 (16.76)	109 (13.68)	68 (26.25)	<.001	2.25 (1.60 ~ 3.17)	0.210	1.47 (0.81 ~ 2.68)
Invasive bacteria							
No	1055 (95.82)	797 (95.45)	258 (96.99)		1.00 (Reference)		
Yes	46 (4.18)	38 (4.55)	8 (3.01)	0.277	0.65 (0.30 ~ 1.41)		
Alcohol use, n(%)							
No	1005 (77.73)	765 (79.19)	240 (73.39)		1.00 (Reference)		1.00 (Reference)
Yes	288 (22.27)	201 (20.81)	87 (26.61)	0.030	1.38 (1.03 ~ 1.84)	0.126	0.57 (0.28 ~ 1.17)
Smoking, n(%)							
No	847 (61.69)	661 (64.55)	186 (53.30)		1.00 (Reference)		1.00 (Reference)
Yes	526 (38.31)	363 (35.45)	163 (46.70)	<.001	1.60 (1.25 ~ 2.04)	0.078	1.88 (0.93 ~ 3.77)
History of tuberculosis,							
No	1052 (74.93)	831 (79.14)	221 (62.43)		1.00 (Reference)		1.00 (Reference)
Yes	352 (25.07)	219 (20.86)	133 (37.57)	<.001	2.28 (1.76 ~ 2.97)	<.001	2.72 (1.60 ~ 4.60)
WBC×10^9/L, n(%)							
<4	1057 (75.72)	832 (79.69)	225 (63.92)		1.00 (Reference)		1.00 (Reference)
4-10	131 (9.38)	106 (10.15)	25 (7.10)	0.560	0.87 (0.55 ~ 1.38)	0.511	1.58 (0.40 ~ 6.16)
>10	208 (14.90)	106 (10.15)	102 (28.98)	<.001	3.56 (2.61 ~ 4.85)	0.363	0.58 (0.18 ~ 1.88)
HGB (g/L), n(%)							
>90	1303 (93.34)	988 (94.64)	315 (89.49)		1.00 (Reference)		1.00 (Reference)
≤90	93 (6.66)	56 (5.36)	37 (10.51)	0.001	2.07 (1.34 ~ 3.20)	0.246	0.53 (0.18 ~ 1.54)
PLT×10^9/L, n(%)							
>100	1358 (97.35)	1022 (97.89)	336 (95.73)		1.00 (Reference)		1.00 (Reference)
<100	37 (2.65)	22 (2.11)	15 (4.27)	0.032	2.07 (1.06 ~ 4.04)	0.881	0.85 (0.11 ~ 6.75)
Lymphocyte×10^9/L, n(%)							

⩾0.8	1166 (83.58)	933 (89.45)	233 (66.19)		1.00 (Reference)		1.00 (Reference)
<0.8	229 (16.42)	110 (10.55)	119 (33.81)	<.001	4.33 (3.22 ~ 5.83)	0.042	1.99 (1.02 ~ 3.88)
Monocyte×10^9/L, n(%)							
⩽0.8	1155 (82.80)	905 (86.77)	250 (71.02)		1.00 (Reference)		1.00 (Reference)
>0.8	240 (17.20)	138 (13.23)	102 (28.98)	<.001	2.68 (2.00 ~ 3.58)	0.030	2.00 (1.07 ~ 3.72)
Neutrophil×10^9/L, n(%)							
2-7	1056 (75.70)	843 (80.82)	213 (60.51)		1.00 (Reference)		1.00 (Reference)
<2	83 (5.95)	74 (7.09)	9 (2.56)	0.043	0.48 (0.24 ~ 0.98)	0.980	0.00 (0.00 ~ Inf)
>7	256 (18.35)	126 (12.08)	130 (36.93)	<.001	4.08 (3.06 ~ 5.44)	0.011	4.39 (1.41 ~ 13.63)
ALT(U/L), n(%)							
≤40	1245 (90.09)	936 (90.43)	309 (89.05)		1.00 (Reference)		
>40	137 (9.91)	99 (9.57)	38 (10.95)	0.455	1.16 (0.78 ~ 1.73)		
AST(U/L), n(%)							
≤40	1211 (87.44)	926 (89.30)	285 (81.90)		1.00 (Reference)		1.00 (Reference)
>40	174 (12.56)	111 (10.70)	63 (18.10)	<.001	1.84 (1.32 ~ 2.58)	0.427	0.71 (0.30 ~ 1.66)
ALP(U/L), n(%)							
≤150	1221 (94.14)	937 (96.00)	284 (88.47)		1.00 (Reference)		1.00 (Reference)
>150	76 (5.86)	39 (4.00)	37 (11.53)	<.001	3.13 (1.96 ~ 5.00)	0.170	2.06 (0.73 ~ 5.78)
TBil(μmol/L), n(%)							
≤20.5	1271 (91.97)	961 (92.76)	310 (89.60)		1.00 (Reference)		
>20.5	111 (8.03)	75 (7.24)	36 (10.40)	0.062	1.49 (0.98 ~ 2.26)		
DBil(μmol/L), n(%)							
≤7	1291 (93.42)	989 (95.46)	302 (87.28)		1.00 (Reference)		1.00 (Reference)
>7	91 (6.58)	47 (4.54)	44 (12.72)	<.001	3.07 (1.99 ~ 4.72)	0.824	1.12 (0.41 ~ 3.11)

Cre(μmol/L), n(%)							
≤104	1329 (96.65)	1000 (96.90)	329 (95.92)		1.00 (Reference)		
>104	46 (3.35)	32 (3.10)	14 (4.08)	0.383	1.33 (0.70 ~ 2.52)		
Na (mmol/L), n(%)							
⩾135	1129 (82.53)	905 (88.55)	224 (64.74)		1.00 (Reference)		1.00 (Reference)
<135	239 (17.47)	117 (11.45)	122 (35.26)	<.001	4.21 (3.14 ~ 5.65)	0.014	2.24 (1.17 ~ 4.27)
K(mmol/L), n(%)							
⩾3.5	1226 (89.55)	937 (91.59)	289 (83.53)		1.00 (Reference)		1.00 (Reference)
<3.5	143 (10.45)	86 (8.41)	57 (16.47)	<.001	2.15 (1.50 ~ 3.08)	0.100	1.83 (0.89 ~ 3.75)
Glucose (mmol/L), n(%)							
⩽6.1	927 (69.23)	728 (72.65)	199 (59.05)		1.00 (Reference)		1.00 (Reference)
>6.1	412 (30.77)	274 (27.35)	138 (40.95)	<.001	1.84 (1.42 ~ 2.38)	0.238	0.65 (0.32 ~ 1.32)
ALB (g/L), n(%)							
≥35	847 (61.33)	737 (71.21)	110 (31.79)		1.00 (Reference)		1.00 (Reference)
<35	534 (38.67)	298 (28.79)	236 (68.21)	<.001	5.31 (4.08 ~ 6.90)	0.006	2.19 (1.25 ~ 3.82)
CRP (mg/L), n(%)							
≤5	317 (27.05)	299 (34.33)	18 (5.98)		1.00 (Reference)		1.00 (Reference)
>5	855 (72.95)	572 (65.67)	283 (94.02)	<.001	8.22 (5.00 ~ 13.50)	0.144	1.83 (0.81 ~ 4.14)

BMI:Body Mass Index; WBC:white blood cell; HGB:hemoglobin; PLT:platelet; ALT:alanine aminotransferase; AST:Aspartate aminotransferase; ALP:Alkaline phosphatase;TBil:Total Bilirubin;DBil:Direct bilirubin; Cre:Creatinine;Na:sodium;K:potassium; ALB:albumin;CRP:C-reactive protein
